# Nitrite Promotes the Growth and Decreases the Lignin Content of *indica* Rice Calli: A Comprehensive Transcriptome Analysis of Nitrite-Responsive Genes during *In Vitro* Culture of Rice

**DOI:** 10.1371/journal.pone.0095105

**Published:** 2014-04-16

**Authors:** Xin Wang, Yang Li, Gen Fang, Qingchuan Zhao, Qi Zeng, Xuemei Li, Hanyu Gong, Yangsheng Li

**Affiliations:** 1 State Key Laboratory of Hybrid Rice, College of Life Sciences, Wuhan University, Wuhan, Hubei, China; 2 CAS Key Laboratory of Plant Germplasm Enhancement and Specialty Agriculture, Wuhan Botanical Garden, Chinese Academy of Sciences, Wuhan, Hubei, China; Institute of Botany, Chinese Academy of Sciences, China

## Abstract

As both major macronutrients and signal molecules, nitrogen metabolites, such as nitrate and nitrite, play an important role in plant growth and development. In this study, the callus growth of *indica* rice cv. 9311 was significantly enhanced by nitrite, whereas the soluble protein content remained unchanged. The deep RNA sequencing technology (RNA-seq) showed that the transcriptional profiles of cv. 9311 calli were significantly changed after adding nitrite to the nitrate-free medium, and these nitrite-responsive genes were involved in a wide range of plant processes, particularly in the secondary metabolite pathways. Interestingly, most of the genes involved in phenylpropanoid-related pathways were coordinately down-regulated by nitrite, such as four cinnamoyl-CoA reductase, and these in turn resulted in the decrease of lignin content of *indica* calli. Furthermore, several candidate genes related to cell growth or stress responses were identified, such as genes coding for expansins, *SMALL AUXIN UP RNA* (*SAUR*) and HSP20s, and these suggested that nitrite could probably serve as a transcriptome signal to enhance the *indica* calli growth by regulation of various downstream genes expression. This study contributes to a better understanding of the function of nitrite during the process of plant tissue culture and could aid in the application of this technology to improved *indica* genetic transformation efficiency.

## Introduction

Rice (*Oryza sativa* L) is one of the most important staple foods, and also a model species for molecular biology and functional genome in gramineae crops. *Indica* varieties account for approximately 70% of the cultivated rice, however, the tissue culture system in this subspecies is mostly specific and *Agrobacterium tumefaciens*-mediated genetic transformation remains difficult [1], [2]. Establishment of a robust and widely applicable culture system for *indica* rice can provide an useful platform for basic biology research, and will also be helpful to develop high-quality cultivars by genetic manipulation [3], [4].

Nitrogen (N) is an essential macronutrient and plays a key role in crop growth and development [5], [6]. As the main source of inorganic nitrogen, nitrate is first reduced to nitrite by nitrate reductase (NR), then to ammonium by nitrite reductase (NiR), and is ultimately incorporated into amino acids. Besides its role as a nutrient, nitrate and its downstream metabolites are known to act as signal molecules to regulate global gene expressions, thus affecting plant physiology and architecture [7]–[9]. Microarray studies showed that the transcriptional profiles had been significantly changed after adding nitrate to the nitrogen-starved *Arabidopsis*, and these nitrate-responsive genes were involved in a wide range of plant processes, such as the nitrate uptake and assimilation process, pentose phosphate pathway and secondary metabolism [10]–[12]. A NR-null mutant of *Arabidopsis* was constructed to indentify a catalogue of NR independent nitrate-responsive genes, which were directly-regulated by nitrate, not downstream metabolites, served as the signal [13]. On the other hand, nitrite, a transient intermediate in the nitrate assimilation, is thought to be toxic metabolite if it is allowed to accumulate in plants. Similar to nitrate, nitrite might also function as a potential signal that regulates various gene expressions [14], [15]. Global transcriptional analysis showed that there was extensive overlap between the nitrate and nitrite-responsive genes, and almost all of the pathways and processes induced by nitrate responded equivalently to nitrite [16].

High-quality embryonic callus is required for the successful *Agrobacterium tumefaciens*-mediated transformation of rice [17]. It has been reported that nitrite is one of determining factors for the callus growth or status in various *japonica* rice. The growth rates of calli were positively correlated with NiR enzyme activities in *japonica*, since the relatively low NiR activities resulted in the accumulation of nitrite and this, in turn, led to browning and inhibited calli growth [18]–[20].

*Indica* cv. 9311 is a widely cultivated variety in China, and is also a typical rice genotype for monocot genomics, whose entire genome sequences have been finished [21]. However, much less information is known about the functions of nitrogen metabolites, especially nitrite, during the process of culture in *indica*. In this study, we have attempted to find that nitrite could be a critical factor for the growth and secondary metabolism of cv. 9311 calli. Comprehensive transcriptome analysis was conducted to indentify the nitrite-responsive genes by use of the deep RNA sequencing technology (RNA-seq). These results contribute to a better understanding of the role of nitrite and could aid in the application of this technology to improved *indica* genetic transformation efficiency.

## Materials and Methods

### Plant materials and culture conditions

Mature seeds of the rice (*Oryza sativa* L. ssp. *indica* cv. 9311) were dehusked and surface sterilized with 70% (v/v) ethanol for 2 min followed by HgCl_2_ 0.1% (v/v) for another 15 min. After five times rinsing with sterile distilled water, the sterilized seeds were placed on LY minimal (or N6 basal) medium, supplemented with 2.5 mg L^−1^ of 2, 4-D, 0.3 g L^−1^ of casein hydrolysate, 3% sucrose and 2.5% phytagel. The cultures were incubated at 30°C under dark condition. Embryonic calli were excised from the scutella of the germinating seeds after 14 days and used for initial materials in this experiment.

### Histological study and protein content measurement

Histology of an embryonic callus was observed according to the method described by Ge et al. [4]. Protein contents of the calli were determined using the BCA assay according to the manufacturer's protocol (Beyotime Biotechnology, China).

### Preparation of cDNA libraries for RNA-Seq

Embryonic calli were inoculated on medium M1 (control, without nitrite) or M2 (with nitrite), see Table 1. At 3 days after inoculation, multiple independent biological replicates, each containing a pool of about 0.5 g fresh weight calli, were harvested and immediately frozen in liquid nitrogen for RNA-Seq (three biological replicates) or quantitative RT-PCR (qRT-PCR) validation (three biological replicates). In our experiment, total RNA of the three independent biological repeats for each sample was isolated using Trizol reagent (Invitrogen). The quality and quantity of the RNA samples were examined by use of the Agilent 2100 Bioanalyzer (Agilent Technologies), and equal amounts of RNA from the three independent biological repeats for each sample were mixed together and then send to Beijing Genomics Institute (BGI, Shenzhen) for libraries construction and sequencing.

**Table 1:** Nitrogen source of medium used in this study.

	Nitrogen source (mM)			
Medium	KNO_3_	KNO_2_	(NH_4_)_2_SO_4_	Glutamine
M1	―	―	3.5	―
M2	―	2	3.5	―
M3	―	―	3.5	20
M4	―	2	3.5	20
N6	28	―	3.5	―

Nitrate was deprived from the N6 medium and 3.5 mM (NH4)_2_SO_4_ was used as the nitrogen source in M1, M2, M3, M4 medium. 2 mM KNO_2_ or 20 mM glutamine was added in these medium if necessary. The other components composed of N6 basal medium (deprive of nitrogen source), 20 mM KCl, 800 mg/L casein hydrolysate, 600 mg/L proline, 2.0 mg/L 2,4-D, 3% sucrose, and 0.3% phytagel at pH 5.8. ‘―’ indicates the component was not added. The standard N6 medium was used as a control in this experiment.

The mRNA was isolated from total RNA using oligo(dT)-magnetic beads and subsequently interrupted to short fragments (about 200 bp) using divalent cations under elevated temperature. Then the first strand cDNA was synthesized by random primer using the mRNA fragments as templates. After second-strand cDNA synthesis and adaptor ligation, cDNA fragments of 200 bp were isolated by gel electrophoresis, and then enriched by PCR amplification. The library products are ready for sequencing analysis via Illumina HiSeq 2000. The deep-sequencing dataset were deposited in the NHI Short Read Archive (accession number: SRR1167032 and SRR1167034).

### Mapping reads to the reference genome and annotated transcripts

Before mapping reads to rice genome (http://rice.plantbiology. msu.edu), it was necessary to filter the dirty raw reads by removing adaptors and low quality reads (the percentage of the low quality bases of quality value≤5 is more than 50% in a read, or over 10% unknown bases in a read). The remaining clean reads were mapped to reference sequences using SOAPaligner/soap2, which allowed no more than 2 bases mismatches in the alignment.

### Normalization of gene expression levels and identification of DGEs

The gene expression was quantified as the count of all reads mapped to the respective genes or loci. The expression level of transcript was normalized by the RPKM value (Reads Per kb per Million reads), which can be directly used for comparing the difference of gene expression within or among samples. The R package DEGseq was performed to identified DGEs from RNA-seq data, with FDR ≤0.001 and the absolute value of log_2_Ratio≥1 as threshold for judgment of significant change [22].

### Validation of RNA-Seq by qRT-PCR

Total RNA was treated with DNase I (Promega) to remove residual genomic DNA and first-strand cDNA synthesis was synthesized using MMLV reverse-transcriptase (Promega). qRT-PCRs were performed in CFX96 Real-Time PCR Detection System (Bio-Rad) using iQ SYBR Green supermix for 40 cycles (95°C for 15 s; 60°C for 15 s; 72°C for 45 s) according to the instruction manual. After the PCR, a melting curve was generated to test the products specificity. Primer sequences and reaction efficiencies were presented in Table S4 online. Three reference genes were selected as the internal control [23]. Data were derived from 3 independent replicates.

### Functional analysis of DEGs based on RNA-Seq data

GO analysis and enrichment were performed using the Singular Enrichment Analysis (SEA) of agriGO (http://bioinfo.cau.edu.cn/agriGO/analysis.php) with the default parameter. This method firstly mapped all DEGs to GO terms in the database and counted the number of genes in every term. Hypergeometric test (with p value < 0.05) was used to find significantly enriched GO terms comparing to the genome background. KEGG pathway analyses of the DEGs were performed using the KEGG Automatic Annotation Server (http://www.genome.jp/tools/kaas/).

### Measurement of nitrite level and lignin content in *indica* calli

About 0.5 g fresh weight calli were homogenized using 1 ml extraction buffer (50 mM Tris-Cl pH 7.5, 5 mM cysteine, 2 mM EDTA-2Na). After centrifuging for 15 min at 12000 rpm (4°C), the crude extracts were used for the determination of nitrite contents, while the sediments were used for the measurement of lignin contents. Briefly, the reaction mixture consisted of 500 µl extracts, 250 µl 0.2% α-naphthylamine and 250 µl 1% sulfanilic acid. After 12 min of incubation at 20°C, the absorbance was recorded at 538 nm. NiR activitiy was measured in a 500 µl reaction mixture containing 425 µl NiR assay buffer (50 mM Tris-Cl pH 7.5, 0.5 mM NaNO_2_, 1 mM methyl viologen), 25 µl enzyme extract and 50 µl start solution (0.12 M Na_2_S_2_O_4_, 0.2 M NaHCO_3_). After 60 min incubation at 30°C, the remaining content of nitrite in the assay mixture was determined by the method above. Lignin content was determined by use of a simple fluorometric method. The sediment was resuspended in 1.5 ml 75% sucrose solution, and fluorescence value of the suspension was detected with excitation wavelength at 360 nm and emission wavelength at 445nm. The amount of lignin was calculated based on the standard curve.

## Results

### Enhancement of callus growth by nitrite in *indica* rice

**Figure 1:**
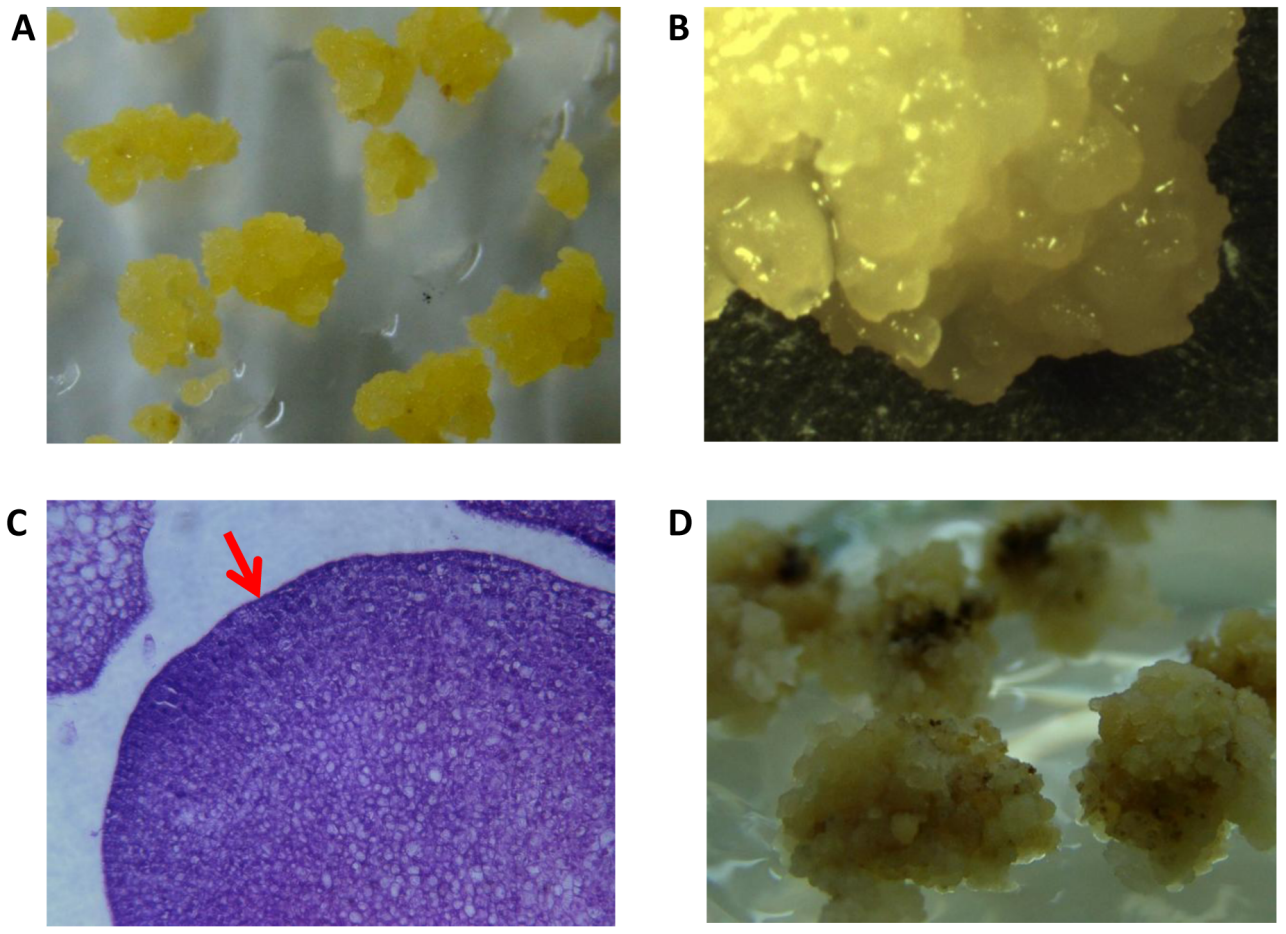
The morphology and histology of cv. 9311 calli. The light yellowish and vigorously growing calli of cv. 9311 after two weeks subculture (A, B); histological view of cv. 9311 calli (C), the embryogenic cells which exhibited dense cytoplasm and small vacuoles were indicated by the red arrow at the surface of calli; the browning calli of cv. 9311 (D).

Many reports have shown that callus induction or subculture is genotype specific in *indica* rice cultivars [2]. In our experiment, it was empirically found that the light yellowish and vigorously growing cv. 9311 calli after two weeks induction were optimal for subculture, and chosen as the initial materials in the study (Figure 1A). These high-quality calli were dry, compact, and globular in appearance (Figure 1B). Histological observation showed that there were clusters of proembryos at the surface of the calli and the cells of proembryos exhibited dense cytoplasm and small vacuoles (Figure 1C), indicating they were probably embryonic calli [4].

**Figure 2:**
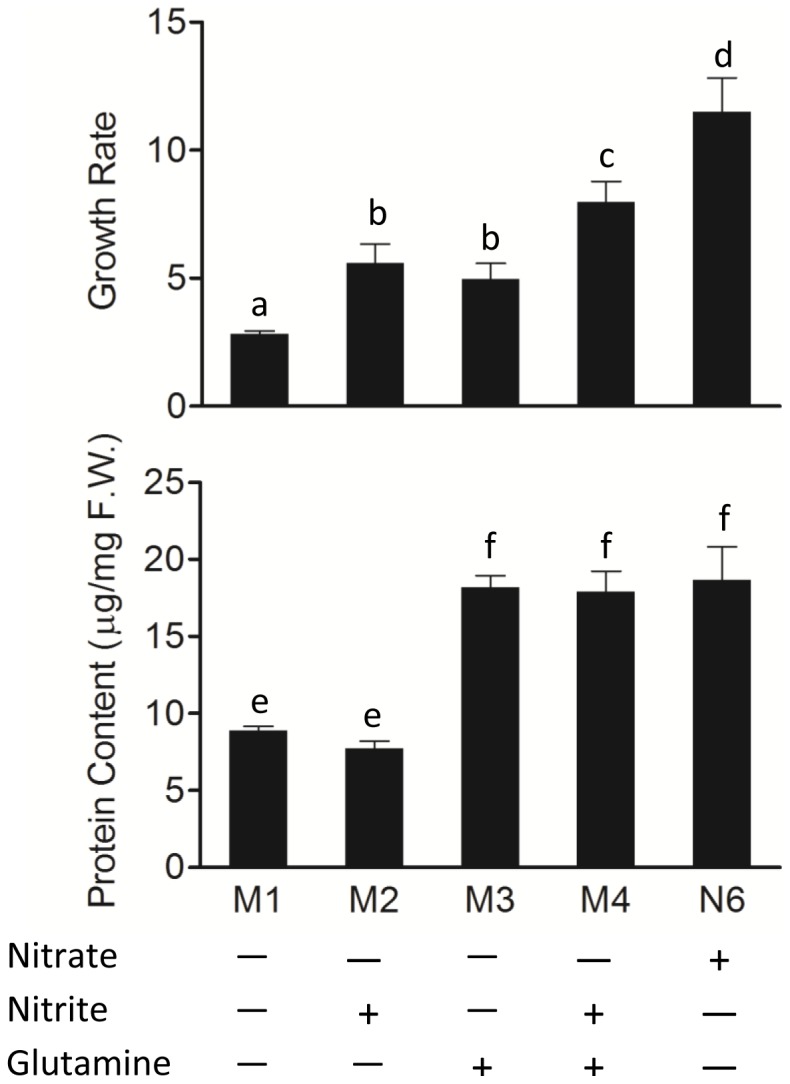
The effect of nitrite on the growth and protein content in cv. 9311 calli under limit or replete nitrogen source. About 0.5(A) and protein content (B) were examined. The growth rates were calculated by calli per flask after culture/amount of calli inoculated. ‘+’ indicates the component was added, while ‘―’ indicates not added, respectively. Data were derived from 3 biological replicates. Means with different letters above the bar indicate a statistical difference between or among the groups (*P<0.05*).

To investigate the nitrite role in the tissue culture process, embryonic cv. 9311 calli were inoculated on the four formulated nitrate-free medium, which was deprived of nitrate and supplied with ammonium [3.5 mM (NH_4_)_2_SO_4_] or 20 mM glutamine as nitrogen source in N6 medium (Table 1). It was found that 2 mM additional KNO_2_ would be optimal for the growth of cv. 9311 calli on this nitrate-free medium, since a higher level of nitrite led to be browning in appearance (Figure 1D). As shown in Figure 2, the cv. 9311 calli inoculated on the M2 (with nitrite) grew significantly faster than those on the M1 (without nitrite), but the soluble protein contents of the calli grown on the two medium remained at similar levels. Moreover, when replete-nitrogen source (20 mM glutamine) was added to the nitrate-free medium (Table 1), the growth rate of calli inoculated on the M4 (with nitrite) was much higher than that on the M3 (without nitrite), and their soluble protein contents were almost at same levels. To exclude the possibility that ammonium was involved in the nitrite response, 1 mM additional (NH_4_)_2_SO_4_ (equal to 2 mM NH_4_^+^) was adding to the M1, and the growth rate of calli was found to have no significant difference with those grown on M1 (data not shown). As both a nutrition and signal molecule, nitrate is an important component of traditional medium for *indica* calli, we also added a standard N6 medium with sufficient nitrate as a control in our experiment. As showed in figure 2, the growth rate of calli inoculated on N6 was much higher than those inoculated on medium without nitrate (M1,M2,M3,M4), but the protein contents remained similar level with those grown on glutamine sufficient medium (M3,M4). This study focused on the function of nitrite during *in vitro* culture of rice. Since nitrate can be converted to nitrite by NR, if we added sufficient nitrate in the medium, it could be converted into nitrite when it was absorbed by calli, that's the reason why we used nitrate-free medium in this experiment. Our results indicated that nitrite might also a critical factor for *indica* calli growth during the nitrate assimilation process, as it could significantly promote the growth of *indica* calli on the nitrate-free medium, although the calli grew more slowly than those grown on nitrate sufficient medium (N6). Taken together, it was concluded that appropriate nitrite could promote callus growth under limit- or replete-nitrogen source condition, and this action was independent of the soluble protein level in *indica* rice.

### Transcriptome analysis of nitrite-response genes using RNA-seq

Nitrite could be regarded as a signal molecule, and induced alterations in genome-wide gene expression in plant cells [16]. To explore the function of nitrite during the process of culture, the deep RNA sequencing technology (RNA-seq) was used to identify the nitrite-responsive genes. RNA-seq can generate absolute information of the global gene expression profiles, rather than relative gene expression measurements; thus, it avoids many of the inherent limitations of microarray analysis [24], [25]. Embryonic calli were incubated on the M1 (control) or M2 (with nitrite) for 3 days, and then two cDNA libraries derived from 9311-1 (control) and 9311-2 (with nitrite) were constructed, sequenced and generated 12,354,248 or 12,015,783 reads, respectively, each of which was 42–50 bp in length (Table 2). The sequence reads were aligned to the rice reference genome database using SOAPaligner/soap2 software (allow 2 base mismatches). Of the total reads, about 78% matched to a unique genomic location, and they were used for further analysis.

**Table 2:** Summary of read numbers based on the RNA-Seq data from rice calli exposed to nitrite.

	9311-1	9311-2
Total reads	12,354,248	12,015,783
Mapped reads	10,129,653 (81.99%)	10,124,245 (84.26%)
Unique match	9,557,782 (77.36%)	9,444,300 (78.60%)
Multi-position match	571,871 (4.63%)	679,945 (5.66%)
Unmapped reads	2,224,595 (18.01%)	1,891,538 (15.74%)

The cv. 9311 calli were inoculated on the M1 or M2 for 3 days, and then two cDNA libraries derived from 9311-1 (control) and 9311-2 (with nitrite), were constructed and sequenced.

**Figure 3:**
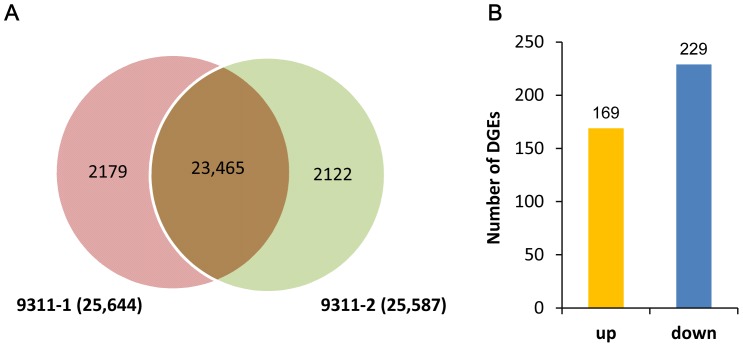
The statistics of differentially expressed genes (DGEs) in the samples. As shown in the venn diagram (A), the number of specifically expressed genes is 2179 (9311-1) and 2122 (9311-2), respectively. Among the 23456 co-expressed genes, 398 are differentially expressed (the absolute value of log_2_(9311-2 vs 9311-1)≥1 and FDR≤0.001), with 169 up-regulated and 229 down-regulated (B).

A total of 25,644 (9311-1), 25,587 (9311-2) genes were detected in the sample. As shown in the venn diagram (Figure 3A), less than 10% of the genes were specific in the library of 9311-1 (2,179) or 9311-2 (2,122), respectively; and the removal of overlapping sequences yielded 27,766 genes, providing abundant data for our further analysis. In addition, the distribution of the length of gene is summarized in Table S1. Particularly, up to 36.5% of the sequences were found to be matched to the genes longer than 2,000 bp, whereas the match efficiency decreased to about 20.44% for those ranging from 500 to 1,000 bp. To facilitate the global analysis of gene function, the detected rice genes were classified into different categories by used of gene ontology (GO) analysis. Of the total annotated genes, 24,460 were assigned to different secondary level GO terms, which belonged to the three main categories of the GO classification (Table S2).

### Global identification of DGEs in response to nitrite

Gene expression levels of 9311-1 or 9311-2 are quantified by the ‘RPKM’ method, which eliminates the influence of different gene length and sequencing discrepancy within or between samples. To indentify nitrite-responsive genes in the cv. 9311 callus, differentially expressed genes (DEGs) between 9311-1 and 9311-2 were screened by a strict algorithm, which was required a twofold or greater changes in expression and false discovery rate (FDR) of 0.001 or less as the threshold. By this approach, we obtained a set of 398 significantly changed genes, with 169 genes up-regulated and 229 genes down-regulated (Figure 3B and Table S3).

The nitrite-responsive DEG profiles detected by RNA-seq were examined for 11 selected genes using quantitative RT-PCR (qRT-PCR) analysis in three independent biological samples cultured under the same conditions. Comparison of RNA-seq and qRT-PCR assay data indicated a similar expression trend, confirming the reliability of the RNA-seq results (Table S4).

### Functional analysis of DGEs

**Figure 4:**
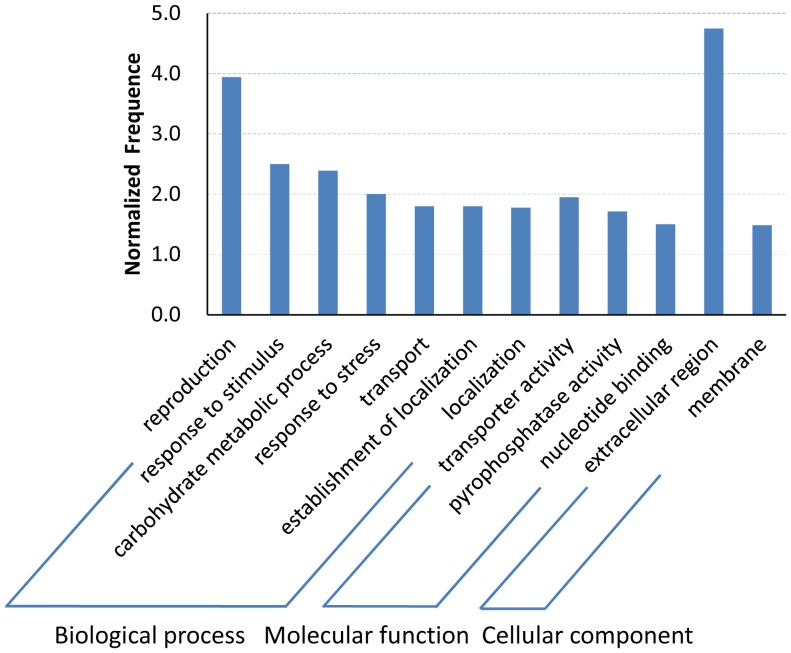
Histogram presentation of enriched gene ontology (GO) classification. All the DEGs were mapped to GO terms in the database, and counted the frequency of genes in every class. The normalized frequency (y-axis) were calculated as frequency of the class in the input data set divided by the frequency of the class in the genome, and the significant enriched GO terms were presented with *p* value <0.05.

To understand the functions of the 398 DGEs, we categorized the genes into 71 secondary level GO terms. In each of the three main categories (biological process, molecular function, and cellular component), there were 36, 20, and 15 secondary level GO terms, respectively (Table S5). Among these groups, a total of 11 secondary level GO terms were overrepresented in the whole data set compared with the reference rice genome data (Figure 4). We found that 6 following Go terms were significantly enriched in the ‘biological process’ category: reproduction (GO: 0000003), response to stimulus (GO: 0050896), carbohydrate metabolic process (GO: 0005975), response to stress (GO: 0006950), transport (GO: 0006810), establishment of localization (GO: 0051234). In terms of ‘molecular function’ category, the enrichment of GO terms were transporter activity (GO: 0005215), pyrophosphatase activity (GO: 0016462) and nucleotide binding (GO: 0000166). Moreover, extracellular region (GO: 0005576) and membrane (GO: 0016020) were dominant in ‘cellular component’ category (Figure 4).

KEGG (http://www.genome.jp/kegg/ko/), a major public pathway-related database, was used for DGEs pathway mapping. Pathway enrichment analysis of the DGEs identified significantly enriched metabolic pathways or signal transduction pathways by comparing these genes with the whole genomic background. Among the 398 nitrite-response DGEs, 213 genes were found with the KEGG pathway annotation. As presented in Table 3, the top 3 enriched pathways were all related to secondary metabolism, including anthocyanin biosynthesis, glucosinolate biosynthesis and phenylpropanoid biosynthesis. Phenylalanine metabolism, the precursors for biosynthesis of phenylpropanoid, was also found to be enriched. In addition, other enriched pathways were observed, such as methane metabolism, starch and sucrose metabolism, plant-pathogen interaction and pyruvate metabolism.

**Table 3:** Pathway enrichment analysis of differential expressed genes.

Pathway Name	DEGs with pathway annotation	All genes with pathway annotation	*P-value*	Pathway ID
Anthocyanin biosynthesis	5 (2.35%)	39 (0.18%)	4.20E-05	ko00942
Glucosinolate biosynthesis	6 (2.82%)	87 (0.41%)	2.42E-04	ko00966
Phenylpropanoid biosynthesis	15 (7.04%)	521 (2.45%)	2.59E-04	ko00940
Methane metabolism	8 (3.76%)	182 (0.86%)	5.13E-04	ko00680
Plant-pathogen interaction	7 (3.29%)	146 (0.69%)	6.88E-04	ko04626
Pyruvate metabolism	6 (2.82%)	128 (0.6%)	1.86E-03	ko00620
Phenylalanine metabolism	9 (4.23%)	275 (1.29%)	1.89E-03	ko00360
Starch and sucrose metabolism	11 (5.16%)	440 (2.07%)	5.00E-03	ko00500

### Coordinate down-regulation of Genes involved in secondary metabolism

Since nitrite had a significant effect on the gene expression of secondary metabolism in *indica* callus, DGEs involved in such processes were further studied. The phenylpropanoid pathway is responsible for the synthesis of lignin as well as an enormous array of flavonoids or anthocyanins in plants [26]. Interestingly, almost all of the DGEs indentified in these pathways were coordinately down-regulated (Table 4, Figure S1). For example, the expression levels of four cinnamoyl-CoA reductase genes (CCR, EC 1.2.1.44), the first enzyme specific to the biosynthetic pathway leading to monolignols, were all depressed by nitrite. In addition, the transcript level of chalcone synthase (CHS, EC 2.3.1.74), a key enzyme of the flavonoid/isoflavonoid biosynthesis pathway, was largely lower than that of control; and four glucosyl transferase genes (LOC_Os04g12960, LOC_Os04g12970, LOC_Os04g12720, LOC_Os11g04860) in anthocyanin biosynthesis were also down-regulated. Moreover, peroxidases were found to have key roles in lignin polymerization, as they could decrease the extracellular lignin formation in tissue cultures by removal of H_2_O_2_ [27], [28]. Candidate peroxidase genes involved in lignin biosynthesis were also indentified in this experiment (Table 4).

**Table 4:** Summaries of differential expressed genes in secondary metabolism.

	Gene ID	Fold change^a^	Up/Down	*P-value*	FDR	Description
Auxin response factor	LOC_Os04g52670	3.76	up	1.7E-09	5.6E-08	OsSAUR21 - Auxin-responsive SAUR gene family member
	LOC_Os06g45950	1.97	up	4.2E-06	8.0E-05	OsSAUR25 - Auxin-responsive SAUR gene family member
	LOC_Os02g04810	1.06	up	2.2E-45	4.3E-43	auxin response factor 5, putative
	LOC_Os05g48870	1.02	up	7.2E-13	4.3E-11	auxin response factor 15, putative
	LOC_Os08g44750	2.88	up	1.1E-10	4.3E-09	auxin-induced protein 5NG4, putative
Cell wall loosening	LOC_Os01g14650	1.92	up	2.1E-05	3.4E-04	expansin precursor, putative
	LOC_Os10g40710	1.71	up	2.1E-20	2.6E-18	expansin precursor, putative
	LOC_Os03g01260	1.05	up	1.1E-05	2.0E-04	expansin precursor, putative
	LOC_Os04g46630	-1.28	Down	0.0E+00	0.0E+00	expansin precursor, putative
Response to stress or stimulus	LOC_Os01g04360	5.14	up	6.9E-10	2.5E-08	hsp20/alpha crystallin family protein, putative
	LOC_Os01g04340	4.56	up	1.4E-06	3.0E-05	hsp20/alpha crystallin family protein, putative
	LOC_Os04g36750	4.27	up	8.7E-10	3.0E-08	hsp20/alpha crystallin family protein, putative
	LOC_Os01g04370	3.48	up	3.3E-16	3.2E-14	hsp20/alpha crystallin family protein, putative
	LOC_Os03g16030	2.73	up	2.6E-11	1.1E-09	hsp20/alpha crystallin family protein, putative
	LOC_Os03g16020	2.31	up	5.3E-10	1.9E-08	hsp20/alpha crystallin family protein, putative
	LOC_Os03g14180	1.71	up	1.0E-05	1.8E-04	hsp20/alpha crystallin family protein, putative
	LOC_Os01g04350	1.21	up	1.0E-07	2.6E-06	hsp20/alpha crystallin family protein, putative
	LOC_Os07g23570	1.85	up	9.7E-09	3.0E-07	cytochrome P450 72A1, putative
	LOC_Os01g43750	1.62	up	2.9E-108	6.8E-106	cytochrome P450 72A1, putative
	LOC_Os10g38600	1.34	up	3.6E-10	1.3E-08	glutathione S-transferase GSTU6, putative

a Fold change indicates ‘log_2_ Ratio(9311-2/9311-1)’.

### Nitrite-inhibition of lignin content

**Figure 5:**
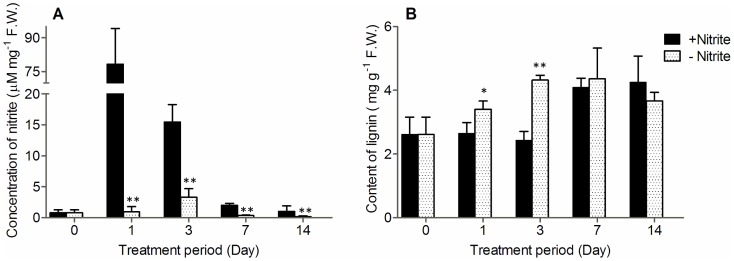
Changes of nitrite level and lignin content in cv. 9311 calli during the process of culture. The cv. 9311 calli were inoculated on the M1 (control, without nitrite) or M2 (with nitrite) for 14 days duration, and sampled at the indicated periods for the measurement of nitrite level and lignin content. Data were derived from 3 independent replicates. Asterisks indicate a statistical difference between calli grown on medium with nitrite (M1) and without (M2, control) at the same inoculated time (*P <0.05, **P <0.001).

As multiple genes assigned to lignin biosynthesis were coordinately repressed after adding nitrite (Table 4, Figure S1), it was proposed that the nitrite level *in vivo* could be negatively correlated with the lignin content. To further confirm this hypothesis, we detected the nitrite levels and lignin contents in cv. 9311 calli which were inoculated on the M1 (without nitrite) or M2 (with nitrite) medium during the process of subculture. As shown in Figure 5, the nitrite level (78.31±15.8 µM mg^−1^ F.W.) was sharply increased by about 96-folds of the original level (0.81±0.44 µM mg^−1^ F.W.) after 1 day inoculation on M2, then decreased to 15.43±2.83 µM mg^−1^ F.W. after 3 days, and was finally recovered to approximately its original level after 14 days. However, the lignin contents of calli were not change during the first 3 days, and then it was found to increase after 7 days (4.09±0.28 mg g^−1^ F.W.) when the nitrite level decreased to the relative low level (2.01±0.29 µM mg-1 F.W.). On the other hand, the nitrite content in calli inoculated on M1 (without nitrite) seemed to have not significantly changed, remaining very low level during the whole process of subculture; while the lignin level started to increase after only 1 day inoculation (3.40±0.25 mg g^−1^ F.W.) and gradually reached its maximum level after 7 days (4.36±0.96 mg g^−1^ F.W.). Taken together, during the early stage of subculture (often before 3 days), the higher level of nitrite *in vivo* could inhibit lignin biosynthesis in *indica* calli, as compared to the control (inoculated on M1). This is consistent with RNA-seq results that most genes in lignin biosynthesis were coordinately down-regulated after adding nitrite to medium for 3 days. Furthermore, in the late stage of subculture (often after 7 days), though the nitrite contents decreased to about the original level (0 day) as medium nutrition was almost exhausted, they were still significantly higher than those of control. However, the lignin contents remained at similar levels to those of control during the same period, which suggested that nitrite could inhibit lignin biosynthesis only if it reached a reasonable threshold level.

### Candidate genes involved in the nitrite-promotion of cell growth

Expansins are a class of proteins that promote cell wall looseing and extension during the plant growth and development [29]. In our data, the expressions of 3 expansion-encoding genes (LOC_Os01g14650, LOC_Os10g40710 and LOC_Os03g01260) were significantly up-regulated by nitrite (Table 5). Auxin coordinates numerous growth and development processes by regulating the expression of hundreds of genes. SMALL AUXIN UP RNA (SAUR) is the largest family of auxin-responsive genes [30]–[32]. In this study, several auxin-responsive genes or factors, such as OsSAUR21 (LOC_Os04g52670) and OsSAUR25 (LOC_Os06g45950), were induced after adding nitrite to the medium (Table 5). Furthermore, HSP20s are a group of small molecular weight heat shock proteins (20 KDa), which can be induced to protect plants from the damage caused by various environmental stresses. It was interesting to found that the expressions of 8 *hsp20s* genes identified in DGEs were all up-regulated in response to nitrite (Table 5).

**Table 5:** Summaries of key differential expressed genes related to cell growth or stress response.

Secondary metabolism pathway	Gene ID	Fold change^a^	Up/Down	*P-value*	FDR	Description
Anthocyanin biosynthesis	LOC_Os04g12960	–2.58	Down	5.1E-14	4.3E-12	UDP-glucoronosyl/UDP-glucosyl transferase
	LOC_Os04g12970	–1.62	Down	0.0E+00	0.0E+00	UDP-glucoronosyl/UDP-glucosyl transferase
	LOC_Os04g12720	–1.72	Down	0.0E+00	0.0E+00	indole-3-acetate beta-glucosyltransferase
	LOC_Os11g04860	–1.05	Down	7.1E-11	2.8E-09	anthocyanin 5-O-glucosyltransferase
	LOC_Os05g08750	1.57	Up	2.8E-05	4.3E-04	UDP-glucoronosyl and UDP-glucosyl transferase
Flavonoid biosynthesis	LOC_Os04g01354	–2.60	Down	2.9E-11	1.2E-09	chalcone synthase
	LOC_Os07g13800	–1.65	Down	1.2E-14	1.1E-12	cytokinin-N-glucosyltransferase
Lignin biosynthesis	LOC_Os02g56460	–2.21	Down	0.0E+00	0.0E+00	Similar to Cinnamoyl-CoA reductase
	LOC_Os02g56680	–1.96	Down	0.0E+00	0.0E+00	Similar to Cinnamoyl CoA reductase
	LOC_Os02g56700	–1.61	Down	0.0E+00	0.0E+00	Similar to Cinnamoyl-CoA reductase
	LOC_Os02g56690	–2.48	Down	1.6E-09	5.5E-08	Similar to Cinnamoyl-CoA reductase
	LOC_Os04g46970	–1.34	Down	0.0E+00	0.0E+00	Coniferyl-alcohol glucosyltransferase
	LOC_Os01g15830	1.10	Up	3.6E-05	5.5E-04	peroxidase precursor
	LOC_Os05g06970	1.47	Up	2.3E-26	3.5E-24	peroxidase precursor
	LOC_Os05g04410	1.47	Up	3.4E-27	5.3E-25	peroxidase precursor
	LOC_Os11g02130	–1.07	Down	8.5E-13	5.0E-11	peroxidase precursor
	LOC_Os03g55410	–1.20	Down	4.6E-06	8.8E-05	peroxidase precursor
	LOC_Os03g25340	–1.29	Down	5.1E-06	9.5E-05	peroxidase precursor
	LOC_Os06g35520	–1.39	Down	6.3E-12	2.9E-10	peroxidase precursor
	LOC_Os03g25300	–1.84	Down	4.1E-05	6.1E-04	peroxidase precursor
	LOC_Os10g17650	–2.71	Down	2.6E-05	4.0E-04	Os10bglu34 - beta-glucosidase homologue, similar to Os3bglu6
	LOC_Os11g24374	2.42	Up	6.3E-07	1.4E-05	OsSCP55 - Putative Serine Carboxypeptidase homologue

a Fold change indicates ‘log_2_ Ratio(9311-2/9311-1)’.

## Discussion

Nitrogen is one of the major macronutrients for higher plants via incorporation into amino acids or nuclear acids [5], [9]. Also, nitrogen metabolites can be served as signal molecule, and be sensed by plants to regulate their development, physiology, and metabolism [7], [33]. In this study, nitrite was found to be a crucial factor for the promotion of cv. 9311 callus growth. Genome-wide gene expression analysis showed that transcriptional profiles in the secondary metabolism were significantly changed by nitrite in *indica* calli. It has been reported that the growth rates of calli were negatively correlated with the nitrite ion contents in most *japonica* varieties, since the toxic nitrite ions could be over accumulated in the poor-growth calli [18]–[20]. However, our results demonstrated that appropriate concentration of nitrite (2 mM KNO_2_) could stimulate the proliferation of *indica* calli, indicating the difference of nitrogen metabolism between the two rice subspecies.

Several potential candidate genes involved in the nitrite-promotion of callus growth were identified by RNA-seq (Table 5). Cell growth was intimately connected with cell wall in plants. Rice expansins were required for enhancing growth by mediating cell wall loosening, as the OsEXP4 protein level was closely correlated with the seedling growth in transgenic plants [34]. The increased expression of 3 expansion encoding genes strongly indicated they might be key regulators in the callus growth. In addition, auxin-mediated cell expansion appeared to be correlated with the expression of SAUR genes. Overexpression of *Arabidopsis* SAUR proteins conferred increased cell expansion, including larger hypocotyl and leaf size; whereas expressing an artificial microRNA targeting multiple members of the SAUR subfamily exhibited reverse phenotype [30]. The significant up-regulation of 2 SAUR gene family members suggested their involvement in the promotion of callus growth. Since the process of plant tissue culture *in vitro* was considered as an environmental stress, the up-regulation of 8 hsp20s genes identified in DGEs would be advantageous for calli to adapt to this stressful condition. Thus, it was proposed that nitrite could probably serve as a transcriptome signal to enhance the *indica* calli growth by regulation of various downstream candidate genes expression. However, the exact physiological role of nitrite involved in this process needs to be further studied.

Phenylpropanoid-related metabolites play important roles in mediating plant responses to biotic and abiotic stress [35], [36]. As presented in Table 5, several genes involved in phenylpropanoid or lignin formation, such as CCR genes, were coordinately down-regulated in response to nitrite. It has been reported that nitrate could inhibit large sectors of phenylpropanoid metabolism by down-regulation of a set of genes in the early steps of the phenylpropanoid biosynthetic pathway, and deficiency of nitrate resulted in accumulation of many phenylpropanoids and enhanced levels of lignin in tobacco [37]. Suppression of CCR gene by RNAi transgenic plants led to decrease in lignin content and redirection of metabolite flow within phenylpropanoid metabolism [38]–[40]. Moreover, microarray data showed that most of nitrate-induced genes or pathways were also induced by nitrite [16]. Thus, it can be inferred that nitrite, resemble to nitrate, could serve as signal molecule and inhibit the lignin content by regulation of several key genes in phenylpropanoid-related biosynthesis. This study is a preliminary research on the function of nitrite during *in vitro* culture of rice, and the exact physiological role of nitrite involved in this process needs to be further studied, thus additional efforts may be needed to strengthen the conclusion.

Compared with other technologies, *Agrobacterium*-mediated transformation system is considered as an ideal tool for crop genetic improvement, because of its high transformation efficiency, low copy number of transgenic integration and relatively low cost [41]. Extensive efforts have been made to improve the frequency of rice transformation [42]–[44], but the transformation efficiency of many *indica* rice cultivars is still low. The results provided in this experiment showed great promise for improving *indica* rice transformation using genetic manipulation of the rice genome. Firstly, the increased growth rate enhanced by nitrite will be helpful to obtain high-quality of *indica* calli, since it efficiently avoids the callus browning and genomic mutation as the result of the long period of culture. Secondly, lignin, a phenolic polymer abundant in cell walls, provides rigidity and structural support to cell wall polysaccharides. The biosynthesis of lignin can be induced after wounding or pathogen attack as a defense response [27], [45]. Therefore, it was suggested that the decrease of lignin content by nitrite in *indica* calli might inhibit plant host defense response stimulated by *Agrobacterium* infection, and thus lead to the enhancement of *Agrobacterium*-mediated genetic transformation. In fact, we have modified the components of medium according to the protocol used in the experiment and designed the new LY medium, which was successfully applied to diverse *indica* rice cultivars for improved transformation efficiency (unpublished data).

## Supporting Information

Figure S1 **Coordinate down-regulation of genes involved in phenylpropanoid biosynthesis by nitrite in cv. 9311 calli.**
(PPTX)

Table S1 Distribution of the gene sequences detected in cv. 9311 callus.
(DOCX)

Table S2 The annotation of the genes in cv. 9311 callus.
(XLSX)

Table S3 Differentially expressed genes in response to nitrite.
(XLSX)

Table S4 qRT-PCR validation of the selected DGEs indentified by RNA-seq.
(XLSX)

Table S5 Go enrichment analysis of DGEs.
(XLSX)
