# Supplementary material for: Nitrite Promotes the Growth and Decreases the Lignin Content of indica Rice Calli: A Comprehensive Transcriptome Analysis of Nitrite-Responsive Genes during In Vitro Culture of Rice
Source: PLoS One. 2014 Apr 16;9(4):e95105. doi: 10.1371/journal.pone.0095105 (PMC3989302; doi:10.1371/journal.pone.0095105)
Supplement: Figure S1 — Coordinate down-regulation of genes involved in phenylpropanoid biosynthesis by nitrite in cv. 9311 calli. (PPTX) [file pone.0095105.s001.pptx]

## Slide 1
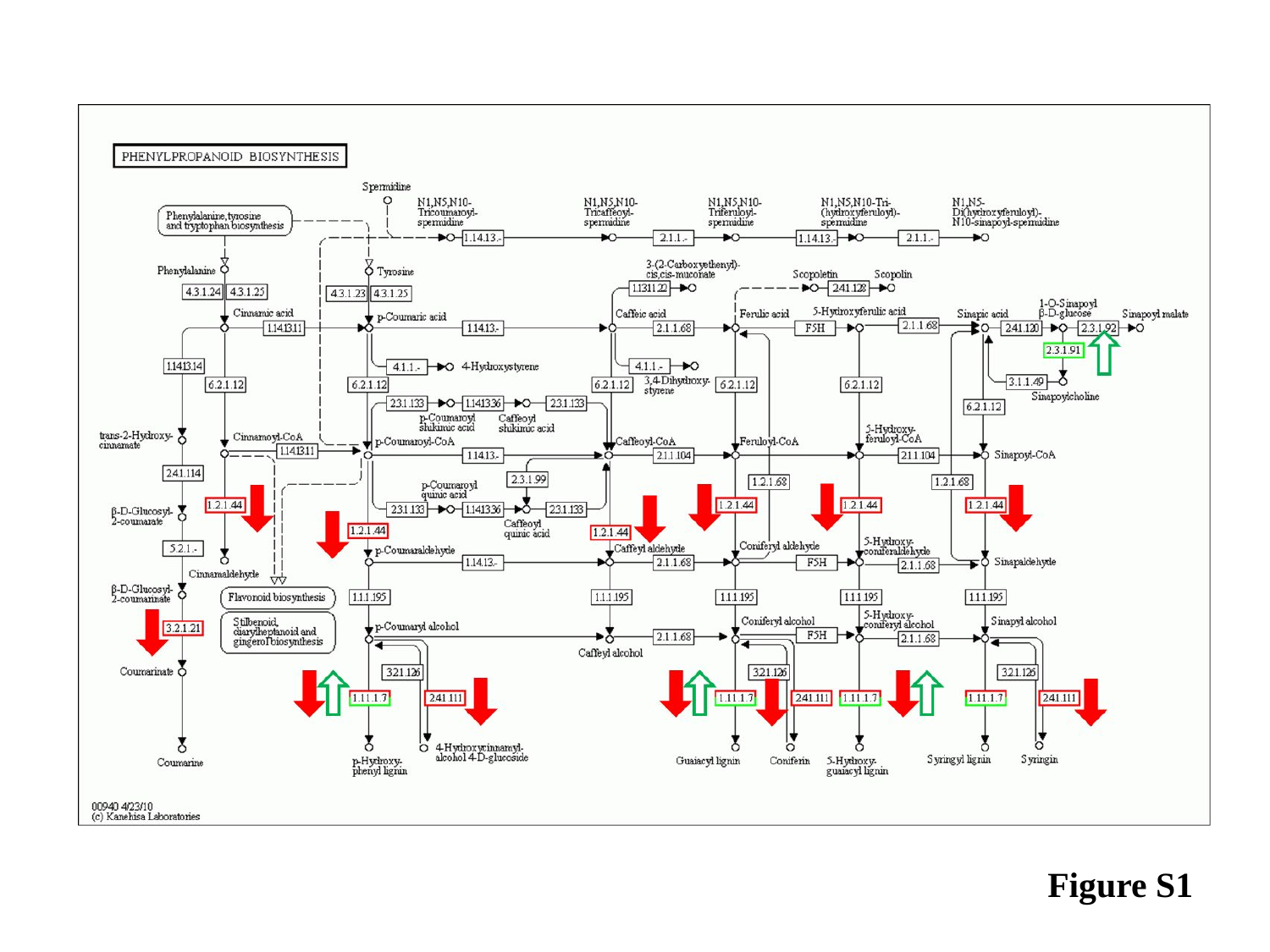

Figure S1

## Slide 2
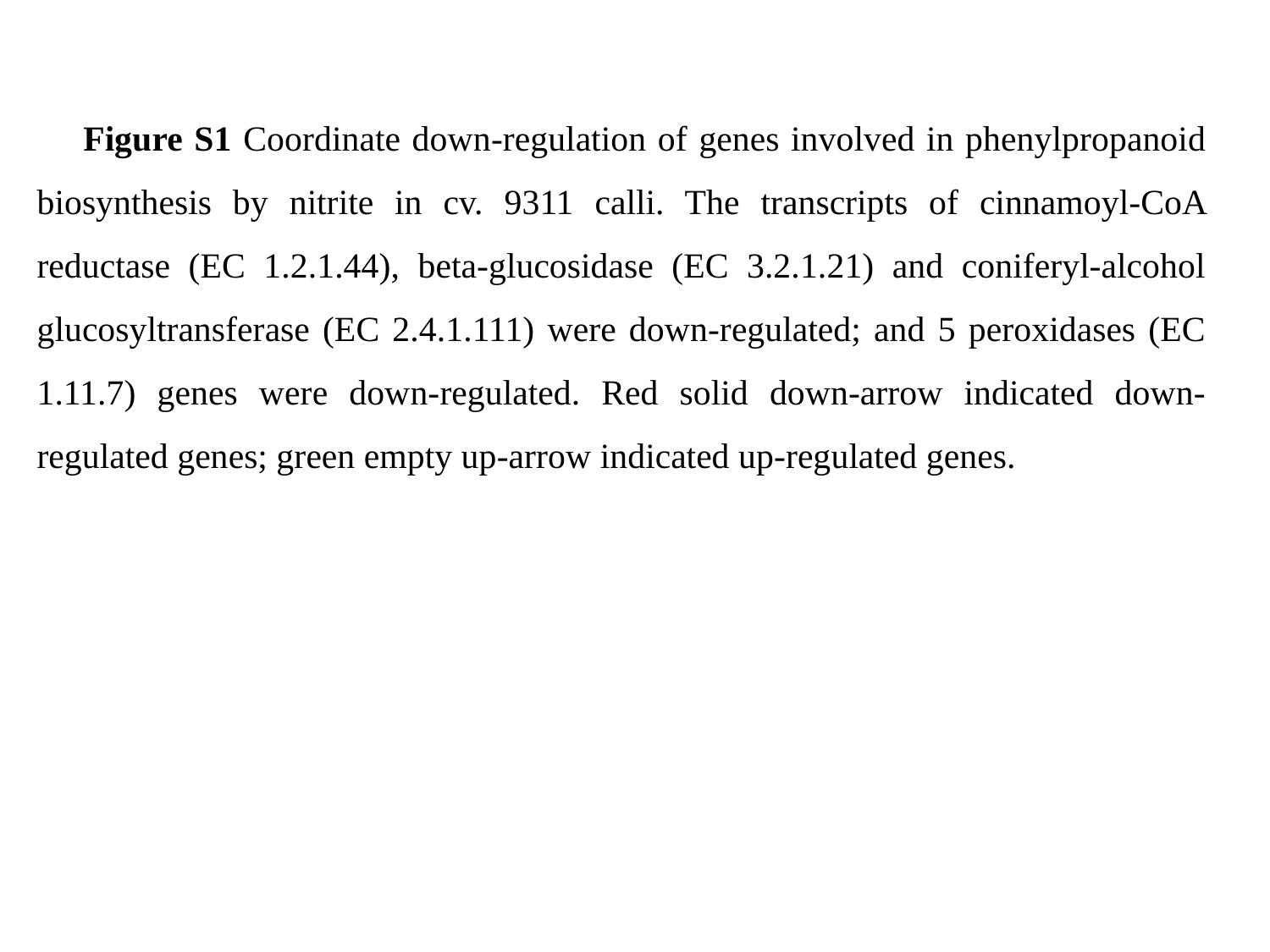

Figure S1 Coordinate down-regulation of genes involved in phenylpropanoid biosynthesis by nitrite in cv. 9311 calli. The transcripts of cinnamoyl-CoA reductase (EC 1.2.1.44), beta-glucosidase (EC 3.2.1.21) and coniferyl-alcohol glucosyltransferase (EC 2.4.1.111) were down-regulated; and 5 peroxidases (EC 1.11.7) genes were down-regulated. Red solid down-arrow indicated down-regulated genes; green empty up-arrow indicated up-regulated genes.
