# Supplementary material for: Nitrite Promotes the Growth and Decreases the Lignin Content of indica Rice Calli: A Comprehensive Transcriptome Analysis of Nitrite-Responsive Genes during In Vitro Culture of Rice
Source: PLoS One. 2014 Apr 16;9(4):e95105. doi: 10.1371/journal.pone.0095105 (PMC3989302; doi:10.1371/journal.pone.0095105)
Supplement: Table S1 — Distribution of the gene sequences detected in cv. 9311 callus. (DOCX) [file pone.0095105.s002.docx]

Table S1 Distribution of the gene sequences detected in rice developing calli exposed to nitrite or without via RNA-Seq.

| Gene length (bp) | Total number | Percentage (%) |
| --- | --- | --- |
| 100-500 | 1576 | 5.68 |
| 500-1000 | 4527 | 16.30 |
| 1000-1500 | 5853 | 21.08 |
| 1500-2000 | 5676 | 20.44 |
| >2000 | 10134 | 36.50 |
| Total | 27766 | 100 |
